# The small heat shock protein αA-crystallin negatively regulates pancreatic tumorigenesis

**DOI:** 10.18632/oncotarget.11668

**Published:** 2016-08-29

**Authors:** Jifang Liu, Zhongwen Luo, Lan Zhang, Ling Wang, Qian Nie, Zheng-Feng Wang, Zhaoxia Huang, Xiaohui Hu, Lili Gong, Andre-Patrick Arrigo, Xiangcheng Tang, Jia-Wen Xiang, Fangyuan Liu, Mi Deng, Weike Ji, Wenfeng Hu, Ji-Ye Zhu, Baojiang Chen, Julia Bridge, Michael A. Hollingsworth, James Gigantelli, Yizhi Liu, Quan D. Nguyen, David Wan-Cheng Li

**Affiliations:** ^1^ State Key Laboratory of Ophthalmology, Zhongshan Ophthalmic Center, Sun Yat-sen University, Guangzhou, Guangdong, 510060, China; ^2^ Department of Ophthalmology & Visual Sciences, Truhlsen Eye Institute, College of Medicine, University of Nebraska Medical Center, Omaha, NE 68198, USA; ^3^ Institute of Cancer Research, The Affiliated Tumor Hospital of Guangzhou Medical College, Guangzhou, Guangdong 510095, China; ^4^ Key Laboratory of Protein Chemistry and Developmental Biology, College of Life Sciences, Hunan Normal University, Changsha, Hunan 410081, China; ^5^ Hepatobiliary Surgery Center of Peking University People's Hospital, Peking University, Beijing 100044, China; ^6^ Department of Biostatistics, College of Public Health, University of Nebraska Medical Center, Omaha, NE 68198, USA; ^7^ Department of Microbiology and Pathology, University of Nebraska Medical Center, Omaha, NE 68198, USA; ^8^ Fred and Pamela Buffett Cancer Center, University of Nebraska Medical Center, Omaha, NE 68198, USA

**Keywords:** small heat shock protein, αA, pancreatic cancer, tumor suppression, cancer therapy

## Abstract

Our recent study has shown that αA-crystallin appears to act as a tumor suppressor in pancreas. Here, we analyzed expression patterns of αA-crystallin in the pancreatic tumor tissue and the neighbor normal tissue from 74 pancreatic cancer patients and also pancreatic cancer cell lines. Immunocytochemistry revealed that αA-crystallin was highly expressed in the normal tissue from 56 patients, but barely detectable in the pancreatic tumor tissue. Moreover, a low level of αA-crystallin predicts poor prognosis for patients with pancreatic duct adenocarcinoma (PDAC). In the 12 pancreatic cell lines analyzed, except for Capan-1 and Miapaca-2 where the level of αA-crystallin was about 80% and 65% of that in the control cell line, HPNE, the remaining pancreatic cancer cells have much lower αA-crystallin levels. Overexpression of αA-crystallin in MiaPaca-1 cells lacking endogenous αA-crystallin significantly decreased its tumorigenicity ability as shown in the colony formation and wound healing assays. In contrast, knockdown of αA-crystallin in the Capan-1 cells significantly increased its tumorigenicity ability as demonstrated in the above assays. Together, our results further demonstrate that αA-crystallin negatively regulates pancreatic tumorigenesis and appears to be a prognosis biomarker for PDAC.

## INTRODUCTION

Pancreatic cancer, the fourth leading cause of cancer related death in the United States among both men and women [1] is one of the few malignancies with high mortality and short median survival period [2]. The high mortality is derived from the fact that most patients are present with metastatic or locally advanced diseases at the time of diagnosis. In addition, the pancreatic cancer cells are resistant to conventional chemotherapy and radiotherapy [3–6].

At the molecular level, pancreatic malignancies are progressed from non-neoplastic cells to invasive adenocarcinoma through a series of pre-malignant lesions characterized by progressively increasing dysplasia. These precursors are named as pancreatic intraepithelial neoplasia (PanINs) with well-characterized stages including PanIN-1a-flat, PanIN-1b-papillary without dysplasia, PanIn-2-papillary with dysplasia, and PanIN-3-carcinoma-*in situ* [4]. Development of pancreatic malignancies is resulted from orchestrated actions of canonical oncogenes and tumor suppressor genes, such as Ki-Ras, p16, p53, Smad4 and BRCA2. The functions of these genes are regulated by various cellular signaling pathways including TGFβ/SMAD, PI3K/AKT, and MAPK pathways [7–17].

αA-crystallin is a member of the small heat-shock protein family (sHSPs) with multiple functions. Small HSPs act as molecular chaperones, and participate in signaling transduction, cell proliferation, cell metabolism, cell survival, apoptosis, senescence, exocytosis and endocytosis [18–22]. Studies from numerous laboratories including ours have revealed that sHSPs also actively regulate tumorigenesis [23–26]. As a major lens structural protein, αA-crystallin is also expressed in non-lenticular tissues including retina, spleen and thymus [27]. Our recent study demonstrated that αA-crystallin is significantly expressed in mouse pancreas [28]. Moreover, analysis of αA-crystallin in the tissue array samples from normal human pancreas and dozens of cases of pancreatic carcinoma reveals significant difference. αA-crystallin is decreased over 10-fold in the pancreatic carcinoma of various types than that in normal pancreas, suggesting that αA-crystallin has tumor suppression functions. Moreover, αA-crystallin negatively regulates cell migration as shown in the pancreatic cancer cell wound healing assay [28].

To further examine if αA-crystallin expression is linked to inhibition of pancreatic cancer development, we have analyzed the expression levels of αA-crystallin in the pancreatic tumor tissue verse the neighboring normal tissues from 74 patients and found that in 56 of 74 patients, expression of αA-crystallin was significantly decreased in the tumor tissue than that in the neighbor tissue. Moreover, we have also examined the expression level of αA-crystallin in various pancreatic cancer cell lines and further tested the role of αA-crystallin in inhibiting cancer development in these cells. Our data show that expression of αA-crystallin is significantly lower in majority of pancreatic cancer cell lines compared with the nestin-expressing normal pancreatic cancer cells (HPNE cells) [29]. When αA-crystallin is knocked down in the pancreatic cells expressing moderate αA-crystallin, the transformation and cell migration abilities are clearly increased. In contrast, when αA-crystallin is expressed in those pancreatic cancer cells lacking endogenous αA-crystallin, the transformation and cell migration abilities of the transgenic cells became significantly decreased. Together, our results support the conclusion that αA-crystallin negatively regulates pancreatic tumorigenesis and decreased expression of αA-crystallin independently predicts poor prognosis of pancreatic cancer.

## RESULTS

### αA-crystallin expression patterns in tissue samples from pancreatic cancer patients

**Figure 1:**
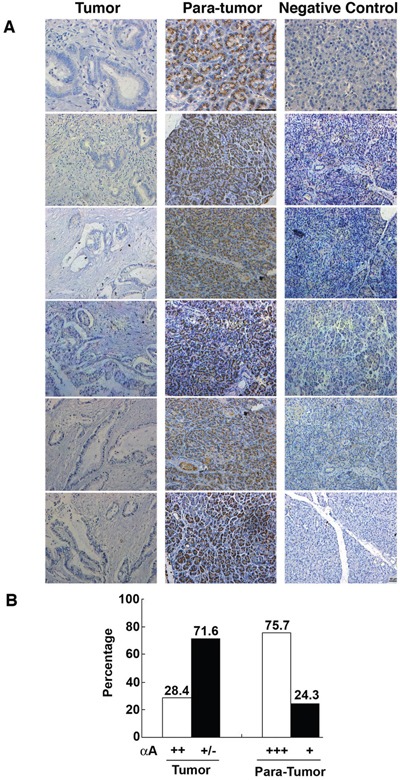
Contrast expression patterns of αA-crystallin in pancreatic cancer tissues and para-tumor tissues **A.** Representative images of immunohistochemical (IHC) assays of αA-crystallin in paired pancreatic tumors and para-tumor tissues. Scar bars, 50μm. **B.** Quantitation of high or low levels of αA-crystallin expression in pancreatic cancer samples and para-tumor tissues.

To further determine the relationship between expression of the αA-crystallin and development of pancreatic cancer, we analyzed the expression patterns of αA-crystallin in 74 paired pancreatic cancer tissues and adjacent non-tumor tissues using immunohistochemistry analysis. As shown in Figure 1, αA-crystallin was localized in the cytoplasm of pancreatic epithelial cells of the para-tumor tissue but hardly detectable in the tumor cells. Quantitation of the positive signals demonstrated that 56/74 (75.7%) adjacent non-tumor tissues displayed strong αA-crystallin expression. In contrast, only 21 of 74 (28.4%) patients exhibited some overexpression of αA-crystallin (scored as <3) in both pancreatic cancer tissues and the adjacent non-tumor tissues. Therefore, αA-crystallin seems to be dramatically decreased during pancreatic carcinogenesis.

### Relationship between αA-crystallin expression and clinical outcome of patients

**Figure 2:**
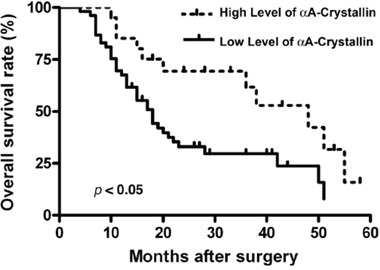
Prognostic significance assessed by Kaplan-Meier survival curves and log-rank tests Comparison of overall survival (OS) according to αA-crystallin expression.

Next, we analyzed the correlation between αA-crystallin expression with clinicopathologic factors of patients with PDAC including gender, age, tumor size, differentiation, pT classification, lymph node metastasis and neural infiltration. As shown in Table 1, a decreased αA-crystallin expression was significantly correlated with pT classification and lymph node metastasis (P=0.019 and P=0.004, respectively), but not with other clinical or pathologic factors. To track the correlation between levels of αA-crystallin and the overall survival (OS) of patents, we followed the patients for 5 years. Survival analysis by the Kaplan-Meier method indicated that OS (P= 0.011) was significantly worse among patients with αA crystallin-low group (Figure 2). Patients in αA-crystallin-low group had less median OS (18 vs 48 months) than those in αA-crystallin-high group. Thus, a low level of αA-crystallin expression was found associated with a poor prognosis of patients with PDAC.

**Table 1:** Associations of αA-Crystallin Expression with Clinicopathological Parameters in 74 PDAC Patients

Characteristics	n	αA-Crystallin Expression		
		High	Low	*p*-value
**Gender**				
**Male**	51	12	39	0.178
**Female**	23	9	14	
**Age (Years)**				0.282
**≤65**	48	15	33	
**> 65**	26	5	21	
**Tumor Size (cm)**				
**≤5**	55	17	38	0.558
**> 5**	19	4	15	
**Differentiation**				0.578
**Well/Moderate**	23	5	18	
**Poor**	51	16	35	
**PT Classification**				0.019
**pT1**	6	4	2	
**pT2**	53	16	37	
**pT3**	15	1	14	
**Lymph Node**				
**pN0**	31	11	20	0.004
**pN1**	43	5	33	
**Neural Infiltration**				
**Yes**	38	9	29	0.442
**No**	36	12	24	

P-values were two-tailed and based on the Pearson chi-square test. *P<0.05* are statistically significant.

Furthermore, multivariate analysis revealed that tumor size, differentiation, pT classification, lymph node metastasis and neural infiltration were unfavorable predictors for OS of PDAC patients, but αA-crystallin was associated with OS (Table 2). Together, a low αA-crystallin level may be used independently to predict poor prognosis for patients with PDAC.

**Table 2:** Multivariate Analyses of Factors Associated with Overall Survival

Variable	OS	
	Hazard Ratio	*p*-Value
Differentiation (well/moderate vs poor)	0.816	0.755
PT classification (pT1/pT2 vs pT3)	1.151	0.817
Lymph node (pN0 vs pN1)	0.754	0.525
Neural infiltration (yes vs no)	0.890	0.801
αA crystallin (high vs low)	2.828	0.017

### mRNA expression of αA-crystallin in various human pancreatic carcinoma cell lines and nestin-expressing pancreatic cells (HPNE)

**Figure 3:**
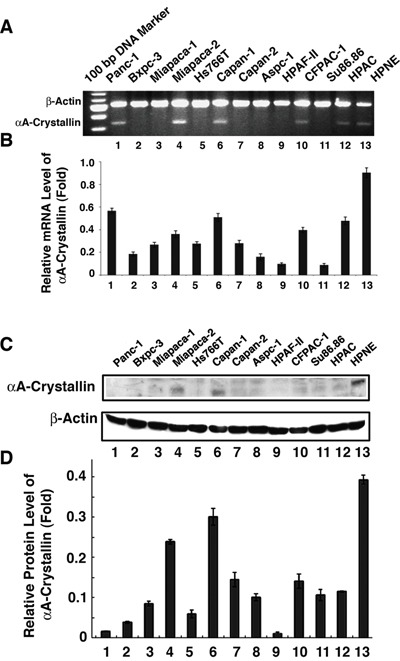
Detection of αA-crystallin mRNA and protein expression in pancreatic cancer cell lines **A.** mRNA levels of αA-crystallin was determined by semi-quantitative RT-PCR assay. Total RNAs were extracted from 12 pancreatic cell lines (Panc-1, Bxpc-3, Miapaca-1, Miapaca-2, Hs766T, Capan-1, Capan-2, Aspc-1, HPAF, CFPAC-1, Su86.86 and HPAC) and HPNE cells (as control), then were used for RT-PCR analyses respectively. **B.** Western blot assays were performed to detect the expression of αA crystallin protein in 12 pancreatic cell lines and HPNE cells. Total proteins were prepared and subjected to western blot assay to determine the expression of αA-crystallin protein. β-actin was used as an internal control. The data shown are representative of three independent experiments. Note that while mRNA was detected in Miapaca-2, Capan-1, Panc-1, CFPAC-1 and HPAC-1, αA-crystallin protein was detected with moderate levels in Miapaca-1 and Capan-1 but much reduced in other pancreatic cancer cell lines in comparison with the control cell, HPNE.

To establish the relative level of mRNA for αA-crystallin in various human cancer cell lines, we have performed RT-PCR analysis. As shown in Figure 3A & 3B, compared with the HPNE cells, various human pancreatic carcinoma cell lines have decreased αA-crystallin mRNA level. Panc-1, Capan-1, FPAC-1, Miapaca-2, and HPAC displayed about 50% to 60%, Bxpc-3, Hs766-T, and the remaining cell lines less than 40% of HPNE αA-crystallin mRNA level.

### Protein expression of αA-crystallin in various human pancreatic carcinoma cell lines and hTERT-pancreas cells

To investigate the relative level of αA-crystallin protein in various human cancer cell lines, we have conducted Western blot analysis. As shown in Figure 3C & 3D, compared with the αA-crystallin level in HPNE cells, various human pancreatic carcinoma cell lines also showed much decreased αA-crystallin level. The two pancreatic tumor cell lines showing the highest αA-crystallin level were Capan-1 and Micpaca-2, which had about 80% and 65% of HPNE αA-crystallin. The remaining pancreatic cancer cell lines had less than 40% of HPNE αA-crystallin.

### αA-crystallin is localized in the cytoplasm and to a less degree in the nucleus of pancreatic tumor cells

To understand the possible function of the αA-crystallin, we have analyzed its localization in pancreatic cancer cells. As shown in Supplementary Figure S1, immunocytochemical analysis revealed that αA-crystallin was largely localized in the cytoplasm and to a much less degree, in the nucleus of pancreatic cancer cells.

### Expression of αA-crystallin in MiaPaCa-1 cells decreases its ability of promoting colony formation

**Figure 4:**
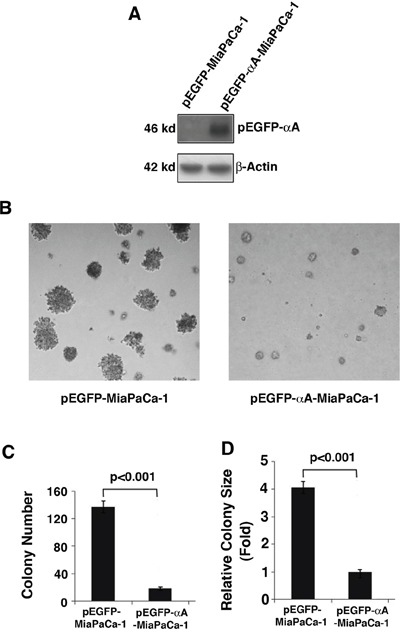
Effects of αA-crystallin expression on cell anchorage-independent growth **A.** Western blot detection of exogenous αA-crystallin in Miapaca-1 cells stably transfected with vector or αA-crystallin. The stable clones were selected using G418 (400 ng/ml) selection. **B.** The stable clones, pEGFP-MiaPaCa-1 or pEGFP-αA-Miapaca-1 were used for the soft agar colony formation assays. Representative cell colonies in soft agar are shown here. **C.** Quantitative analyses of colony numbers and sizes shown in Figure B panels. Values are the means ±SD from three independent experiments.

To analyze the role of αA-crystallin in suppressing pancreatic cancer, we expressed exogenous αA-crystallin in MiaPaCa-1 cells (Figure 4A), which have very little endogenous αA-crystallin and then analyzed the ability of promoting colony formation of these cells. As shown in Figure 4B, 4C & 4D, MiaPaCa-1 cells expressing αA-crystallin displayed significant decrease in both colony size and colony number (about 3-fold decrease). These results support that αA-crystallin has tumor-suppression functions.

### Silence of endogenous αA-crystallin in Capan-1 cells increases its ability of promoting colony formation

**Figure 5:**
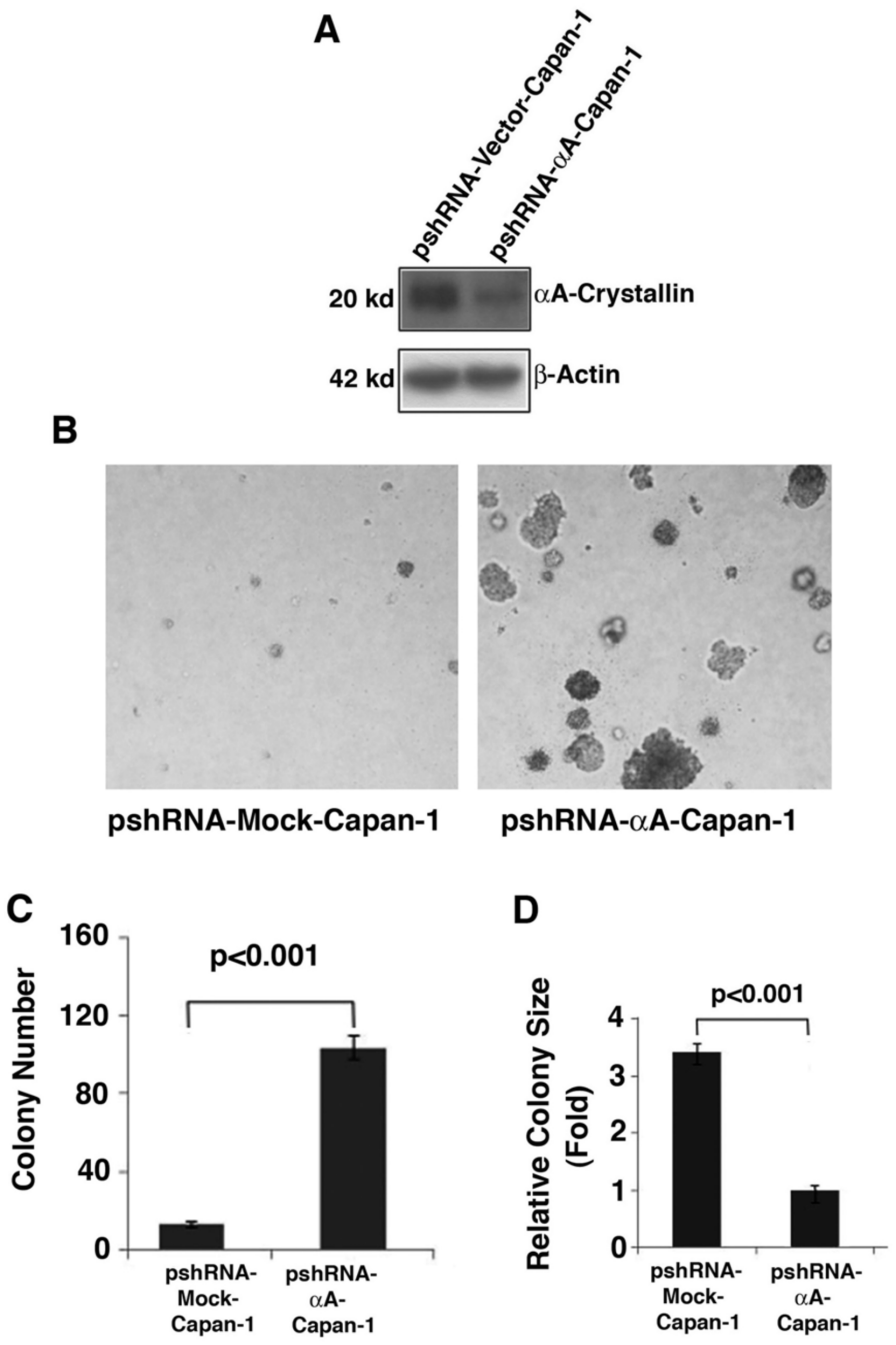
Effects of αA-crystallin silence on cell anchorage-independent growth **A.** Western blot detection of endogenous αA-crystallin in Capan-1 cells stably transfected with control silence vector, pshRNA-mock; or αA-crystallin silence expression vector, pshRNA-αA and selected with purimycin (10 ng/ml). **B.** The stable clones, psh-Mock-Capan-1 or psh-αA-Capan-1 were used for the soft agar colony formation assays. Representative cell colonies in soft agar are shown here. **C.** Quantitative analyses of colony numbers and sizes shown in Figure B panels. Values are the means ±SD from three independent experiments.

To further confirm the role of αA-crystallin in the suppression of pancreatic cancer, we silenced αA-crystallin in Capan-1 cells (Figure 5A), which had relatively moderate endogenous αA-crystallin and then analyzed the ability of promoting colony formation of these cells. As shown in Figure 5B, 5C & 5D, Capan-1 cells with silenced endogenous αA-crystallin exhibited significant increase in both colony size and colony number (about 5-fold increase). These results further support that αA-crystallin has tumor-suppression function.

### Decreased cell migration in αA-crystallin-expressing MiaPaCa-1 cells

**Figure 6:**
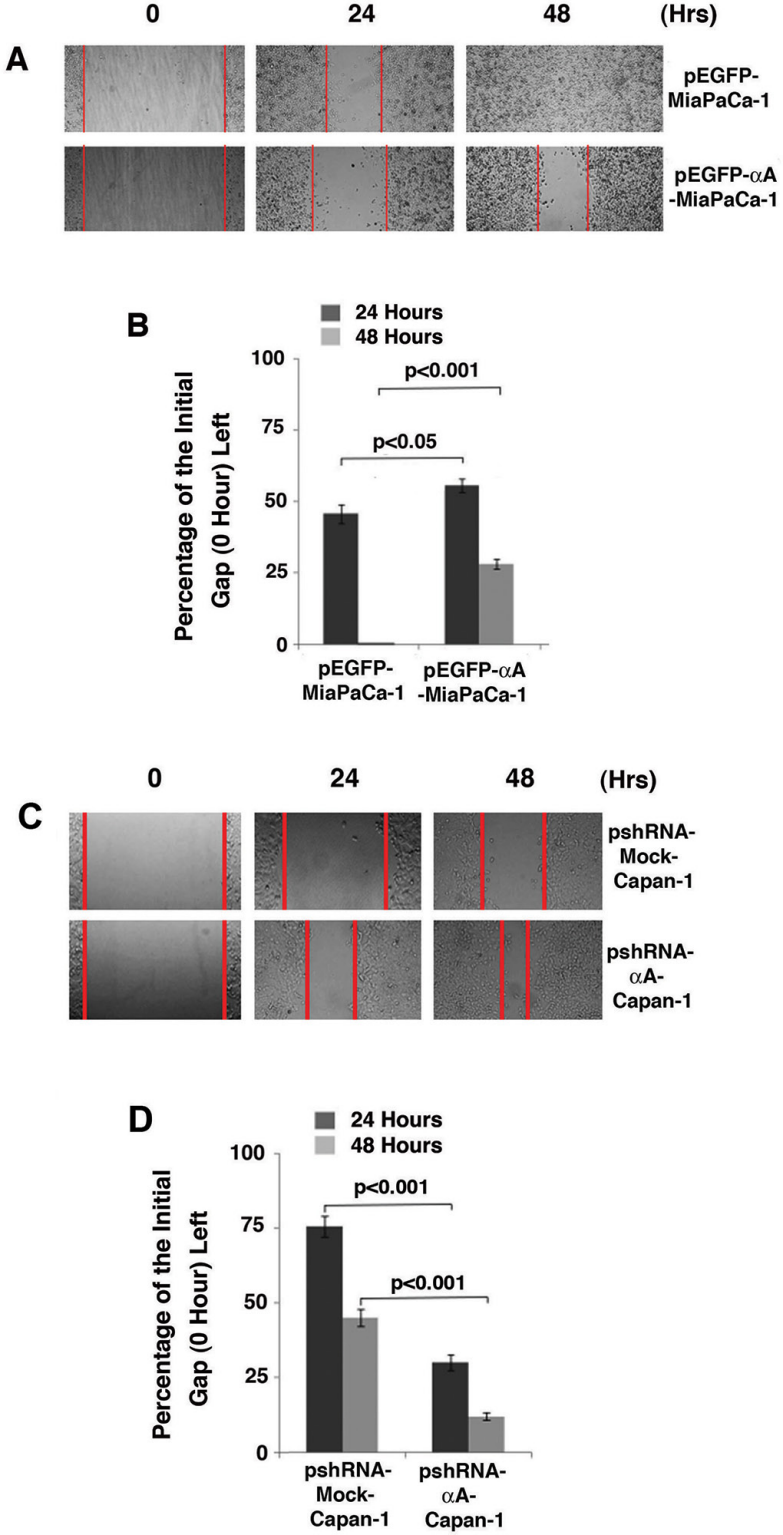
Up-regulation of αA-crystallin retards cell migration A & B. and silence of αA-crystallin promotes cell migration C & D A. Wound healing assay was performed on monolayers of Miapaca-1 cells stably transfected with control vector, or αA-crystallin expression vector. The Representative results were recorded at 0, 24 h and 48 h after wounds were made. 0 h was considered as 100% gap. B. The distance of the wound was measured at 24 and 48 hours along the scratch wound. Values are the means±SD from three independent experiments. C. Wound healing assay was performed on monolayers of Capan-1 cells stably transfected with psh-mock-vector, or psh-αA-crystallin. The Representative results were recorded at 0, 24 h and 48 h after wounds were made. 0 h was considered as 100% gap. D. The distance of the wound was measured at 24 and 48 hours along the scratch wound. Values are the means±SD from three independent experiments.

To explore how αA-crystallin may regulate carcinogenesis, we conducted wound healing assays using the established stable cell lines: pEGFP-MiaPaCa-1 and pEGFP-αA-MiaPaCa-1. As shown in Figure 6A & 6B, MiaPaCa-1 cells expressing αA-crystallin displayed statistically significant inhibition in cell migration. In contrast, the same cells expressing EGFP-vector did not show such effect. Thus, αA-crystallin also regulates migration of pancreatic cancer cells, which is consistent with its decreased expression in various types of pancreatic carcinoma (Figure 1).

### Increased cell migration in αA-crystallin-silencing Capan-1 cells

Since expression of exogenous αA-crystallin in MiaPaCa-1 cells retards its cell migration in the wound healing assay, we next conducted wound healing assay using the established stable cell lines: vector-Capan-1 and αA-crystallin siRNA plasmid-transfected Capan-1 cells. As shown in Figure 6C & 6D, Capan-1 cells expressing αA-crystallin shRNA displayed statistically significant increase in cell migration. In contrast, the same cells expressing vector did not show such effect. Thus, the tumorigenicity ability of capan-1 cells is also dependent upon the level of the endogenous αA-crystallin.

## DISCUSSION

The αA-crystallin and αB-crystallin are initially known as lens structural proteins with about 60% identity in amino acid sequence with each another [30–31]. Several lines of evidence have shown that although the two genes encoding αA- and αB-crystallins seem to arise from gene duplication, they have diverged significantly [32]. First, during mouse development, the two genes are initially turned on at different time. While αB mRNA is first becoming detectable at E9.5, expression of αA mRNA appears at E10.5 [32]. Such differential temporal patterns reflect the differential control mechanisms of the two α-crystallin gene promoters. Second, the two genes display distinct tissue-specific expression patterns. While αA is highly restricted to lens during mouse embryonic developmental process, αB-crystallin is expressed in the developing heart, nasal epithelium, and retinal pigment epithelium [33–35]. In the adult vertebrates, although both αA and αB are abundantly expressed in the lens, they display significantly difference in non-lenticular tissue expressions. αB is strongly expressed in heart, skeletal muscle, kidney and brain [34–35]. In contrast, αA is reported to be expressed at very low level in some non-lenticular tissues including spleen, thymus, heart, brain and liver [35–36]. Our recent studies have shown that αA is also moderately expressed in normal human and mouse pancreases besides its low level of expression in kidney and liver [28].

Existing evidence suggests both αA- and αB-crystallins are implicated in carcinogenesis, yet contrast functions have been detected. Iwaki and Tateishi [37] first demonstrated the existence of αB-crystallin in hamartomas of tuberous sclerosis. Then, it was found that concentrations of αB-crystallin in prostatic carcinoma tissues were significantly higher than in benign prostatic hyperplasia [38]. The same group showed that both αB-crystallin and Hsp27 could be immunohistochemically localized in the normal kidney and renal cell carcinoma tissues. In breast cancer cells, αB-crystallin was found expressing constitutively in certain breast carcinoma cell lines, including those that were capable of metastasizing in immunodeficient mice [39]. Expression of αB-crystallin was associated strongly with lymph node involvement, and to a lesser degree, with high nuclear grade [40]. Increased intensity of αB-crystallin expression was correlated with shorter survival [40]. More recently, αB-crystallin was found commonly expressed in basal-like tumors and its expression predicted poor survival in breast cancer patients independent of other prognostic markers [41]. Moreover, expression of αB-crystallin results in transformation of immortalized human mammary epithelial cells, induction of EGF- and anchorage-independent growth, and enhancement of cell migration and invasion [41]. Thus, αB-crystallin seems to be a novel oncoprotein expressed in basal-like breast carcinomas that independently predicts shorter survival [41]. In addition, high level of αB-crystallin was found contributing to the progression of osteosarcoma [42].

Both αA- and αB-crystallins belong to the heat shock protein (Hsp) family [21]. Compared with αB-crystallin, the limited distribution of αA-crystallin in non-lenticular tissues may restrict its function in carcinogenesis [22–23, 28, 36, 43–44]. Nevertheless, several recent studies suggest that αA-crystallin may be also implicated in tumor development. First, in the noncancerous eyelid, both crystallins were weakly and homogenously expressed in the meibomian gland lobules. However, in human sebaceous carcinoma of the eyelid, both αA-crystallin and αB-crystallin were highly expressed in a few cases examined. A statistically significant correlation was observed between expression levels of the two alpha-crystallins in sebaceous carcinomas [43]. Second, in the retinocytoma, αA-crystallin was expressed in the cytoplasm of all tumor cells, whereas αB-crystallin immunoreactivity was only weakly positive [35]. These results suggest that αA-crystallin, acting like αB-crystallin, seems to promote carcinogenesis. On the other hand, in a recent study where 6 cases of retinoblastoma were subjected to preoperative chemotherapy which induced strong expression of Hsp27 and αB-crystallin but not αA-crystallin [44]. Moreover, the viable tumor cells survived contained high levels of Hsp27 and αB-crystallin but not αA-crystallin. Therefore, these results indicate that αA-crystallin does not seem to follow the same pattern as Hsp27 and αB-crystallin in promoting carcinogenesis. Our recent studies that in 60 different cases of tissue array samples of pancreatic carcinoma, the expression level of αA-crystallin was consistently decreased than that in 11 normal human pancreas samples also support the inhibition of carcinogenesis by αA-crystallin [28]. In the present study, we demonstrated that expression of αA-crystallin in the para-tumor tissues are significantly stronger than that in pancreatic cancer tissues in 56 out of 74 patients. Thus, while αB-crystallin seems to promote tumorigenesis in prostate and breast cancers, αA-crystallin acts as a tumor suppressor against pancreatic cancer development.

Previous studies have shown that numerous factors may be used as prognosis biomarkers for pancreatic cancer [45–54]. These include receptors, kinases and signaling component [45–48], microRNAs or long non-coding RNAs [49–50], serum factor [51], genome sequence or epigenetic status [52–53]. More recently, it was found that the house keeping gene product, α-tubulin, could also act as a prognostic biomarker for pancreatic cancer [54]. Our present studies suggest that αA-crystallin, a small heat shock protein, could also act as a prognostic biomarker for pancreatic cancer. First, compared with its physiological level in normal human pancreas, αA-crystallin is significantly decreased in 60 cases of pancreatic carcinomas of various types [28]; Second, our present studies show that the expression level of αA-crystallin in the para-tumor tissues is much stronger than that in the tumor tissues. In contrast, only 21/74 tumor tissue samples displayed some overexpression of αA-crystallin. Finally, when the level of αA-crystallin expression was correlated with the overall survival (OS), it was found that patients with lower level of αA-crystallin expression had less median OS than those with higher level of αA-crystallin expression (18 vs 48 months, Figure 2). Thus, a low level of αA-crystallin expression in pancreatic cancer seems to predict the poor prognosis of patients with PDAC.

Our results also show that in various pancreatic cancer cell lines examined, only two cell lines, Capan-1 and MiaPACA-2, have detectable αA-crystallin in comparison with the normal HPNE cells (Figure 3). Capan-1 was derived from the liver metastasis of a 40-year-old male with PDAC in the head of the pancreas [55]. On the other hand, MiaPaCa-2 was obtained from a 65-year-old male of PDAC with a palpable upper abdominal mass. The tumor involved the body and tail of the pancreas and had infiltrated the periaortic area [56]. Phenotypically, both cell lines can bind to type I collagen [57–60], have similar invasive properties as tested in Matrigel [38, 60–61], but display differential expression level of COX-2 [62–65], and tumorigenicity ability as assayed in xenograted animal [66–68]. Genetically, both cell lines have mutations in Kras [69] and p53 [69–70], homozygous deletions in p16 [70–71]. However, they have contrast genetic background in Smad 4. While Capan-1 has a mutated gene [72–73], MiaPaCa-2 has a wild type Smad4 gene [69–71, 74]. Regardless their similarity and differences in their phenotype and genetic background, our results demonstrated that knockdown of the endogenous αA-crystallin in both cell lines significantly increases their tumorigenicity (Figures 5 & 6, and data not shown). On the other hand, expression of αA-crystallin in MiaPaCa-1 and another pancreatic cell line, Capan-2 lacking endogenous αA-crystallin significantly decreased their tumorigenicity as tested in the colony formation and wound healing assays (Figures 4A & Figure 6, and data not shown). Together, out results demonstrate that αA-crystallin negatively regulates pancreatic tumor development. Lack of αA-crystallin expression in pancreas may be part of the mechanisms initiating development of pancreatic cancer. We are currently characterizing the exact mechanisms by which αA-crystallin suppresses pancreatic tumorigenesis.

## MATERIALS AND METHODS

### Reagents and antibodies

Anti-αA-crystallin antibody was kindly provided by Dr. Joel Horvitz (University of California at Los Angeles). Human normal pancreas cell line, HPNE [29], was provided by Dr. Michel Ouellette (University of Nebraska Medical Center). All pancreatic carcinoma cell lines [75–87] were provided by Dr. Min Li (University of Oklahoma Cancer Center).

### Patients and tissue samples

The specimens including tumor and matched adjacent non-tumor tissues that were obtained from 74 patients with PDAC who underwent surgical pancreatic resection without preoperative anticancer treatment at the Cancer Center of Guangzhou Medical University and Southern Medical University between May 2005 and June 2010. Ethical approval for the human subjects was obtained from the Ethics Committees of both universities and informed consent was provided to all patients who were followed for 5 years for complete clinical data. Detailed clinical and pathological parameters are summarized in Table 1. Tumor samples were confirmed by histologists in the hospital and were staged according to the TNM classification system endorsed by the World Health Organization. Overall survival (OS) was computed from the day of surgery to the day of death or to the last follow-up.

### Immunohistochemistry

Formalin-fixed tissues were paraffin-embedded and sectioned for immunostained with anti-αA-crystallin antibody using standard immunohistochemistry procedures as previously described [88–97]. Immunostained slides were evaluated independently by 2 pathologists in double-blind manner. Sections were scored semi-quantitatively for the extent of immunoreaction as follows: 0, 0% immunoreactive cells; 1, <5% immunoreactive cells; 2, 5–50% immunoreactive cells; and 3, >50% immunoreactive cells. Also, the intensity of staining was scored semi-quantitatively as following: 0, negative; 1, weak; 2, intermediate; and 3, strong. The final immunoreaction score was defined as the sum of both parameters (extension and intensity). The final scores of <3 were considered to be low in αA crystallin levels, and scores ≥ 3 were considered to be high in αA-crystallin expression.

### Cell lines, establishment of stable cell lines and cell culture

Twelve pancreatic cancer cell lines (Panc-1, Bxpc-3, Miapaca-1, Miapaca-2, Hs766T, Capan-1, Capan-2, Aspc-1, HPAF-II, CFPAC-1, Su86.86 and HPAC) [75–87] were analyzed. The immortalized human pancreatic nestin-expressing cells (HPNE) was kindly provided by Dr. Michel Ouellette (University of Nebraska Medical Center) [29]. The αA cDNA was amplified by RT-PCR from human lens mRNA using the following primers: 5′-TACCTCGAGATGGA-CGTGACCATCCAGC-3′ (αA-crystallin, forward), 5′-CAACCCGGGTTAGGAC-GAGGGAGCCGAG-3′ (αA-crystallin, reverse). The cDNA was further inserted into an enhanced green fluorescence protein expression vector, pEGFPC3, at the XhoI and SmaI sites that were created by PCR to generate in frame fusion construct. The psiRNA-Vector and psiRNA-αA knockdown constructs were ordered from Santa Cruz Biotechnology (CA). The stable transfected cell clones, pEGFP-Miapaca-1 and pEGFP-αA-Miapaca-1, were selected in the presence of 400 μg/ml neomycin in Dulbecco's Modified Eagle's Minimal Essential Medium (DMEM) containing 10% fetal bovine serum, 50 units/ml penicillin and streptomycin as described before [28]. The stable knockdown clones, psiRNA-Mock-Capan-1 and psiRNA-αA-Capan-1 were screened through growth with 0.25 μg/ml puromycin in DMEM containing 10% fetal bovine serum, 50 units/ml penicillin and streptomycin for a period of 4 weeks. All cells were kept at 37°C and 5% CO2 gas phase.

### RT-PCR analysis

The expression level of the mRNA for alphaA-crystallin in human normal and pancreatic carcinoma cell lines were detected using RT-PCR as previously described (28, 88-97). RNA extraction was conducted with RNAeasy kit (Invitrogen). Reverse transcription was conducted with 450 ng total RNA and oligo(dT) primers (Promega). The oligonucleotide primers synthesized by Invitrogen, Inc. were as follows: for alpha A, 5′-ATGGACGAGAAGGTGTTC-3′ (forward) and 5′-TAACGAACCTTAAG-AGCTAC-3′ (reverse) with the amplified fragment of 310 bp; and for human β-actin, 5′-ACATGGCATTGTTACCAAC-3′ (forward) and 5′-CGTTGCCAATAGTGA-TGAC-3′ (reverse) with the amplified fragment of 541 bp. PCR was run 30 cycles with an annealing temperature of 50°C.

### Western blot analysis

Preparation of total proteins from parent and various transfected cells and Western blot analysis of different protein samples were conducted as previously described [88–97].

### Colony formation assay

αA-crystallin knockdown or over-expression cells or control cells were suspended in a medium containing 0.33% agar and overlaid on 0.5% agar in 6-well plates (500 cells/well) as described before [28, 98]. After 14 days, colonies were counted and photographed. The results were expressed as the means ±SD of triplicate counts.

### Wound healing assay

Four types of stable clones of pEGFP-Miapaca-1, pEGFP-αA-Miapaca-1, psiRNA-Vector-Capan-1 and psiRNA-αA-Capan cells-1 [28] were seed in 6-well plates and cultured until 100% confluent. A straight scratch was made by using a 1 ml blue pipette tip to simulate the wound in each well. After PBS washing for 2 times, new DMEM medium was added for a continuous growth of another 48 hours. The wound healing process was recorded daily using the Leica Fluorescence Microscopy with a 10x objective as described before [28, 98].

### Statistical analysis

The student *t*-test was used to compare the mean of two unpaired groups. *P* < 0.05 was considered significant [28, 98]. The Chi-square test was used to study the association between two categorical variables. A Kaplan-Meier plot and log rank test were used to study the association between the overall survival of patients and the expression of αA-crystallin. The Cox proportional hazards regression model was used to study the association between the overall survival of patients and the expression of αA-crystallin by adjusting for other potential confounders.

## SUPPLEMENTARY MATERIALS FIGURE


