# Supplementary material for: The small heat shock protein αA-crystallin negatively regulates pancreatic tumorigenesis
Source: Oncotarget. 2016 Aug 29;7(40):65808–24. doi: 10.18632/oncotarget.11668 (PMC5323194; doi:10.18632/oncotarget.11668)
Supplement: Supplementary file 1 [file oncotarget-07-65808-s001.pdf]

The small heat shock protein αA-crystallin negatively regulates pancreatic tumorigenesis

SUPPLEMENTARY FIGURE

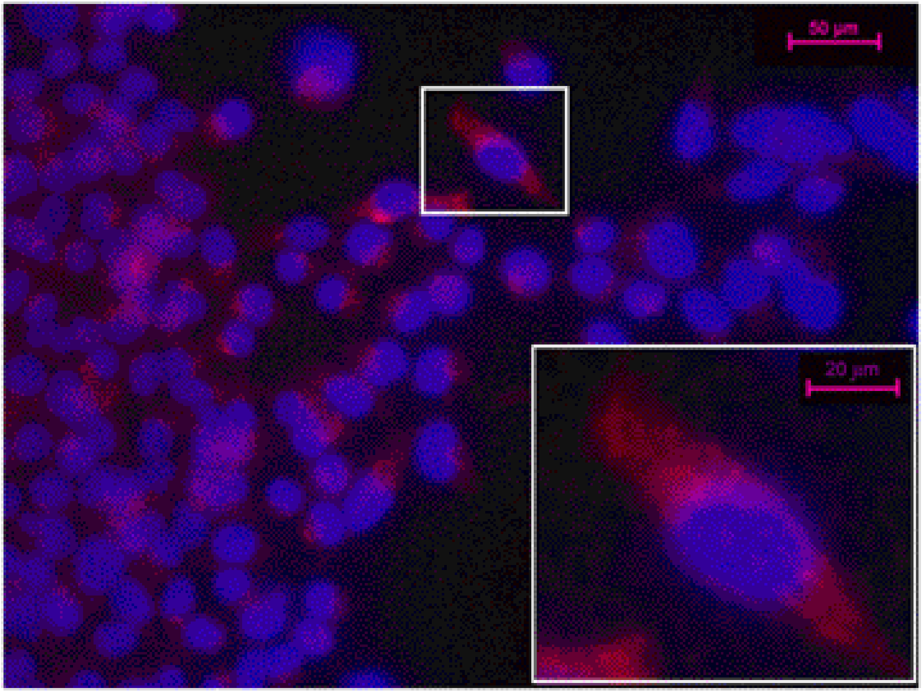

Supplementary Figure S1: Localization of αA-crystallin in Miacapa-2 cells using immunocytochemistry as described before [28, 96–97].
